# Visual perceptions of male obesity: a cross-cultural study examining male and female lay perceptions of obesity in Caucasian males

**DOI:** 10.1186/s12889-015-1821-3

**Published:** 2015-05-16

**Authors:** Eric Robinson, Pleunie S. Hogenkamp

**Affiliations:** Psychological Sciences, University of Liverpool, Liverpool, UK; Department of Neuroscience, University of Uppsala, Uppsala, Sweden

**Keywords:** Obesity, Normalization, Weight loss attitudes, Weight misperceptions, Body size perception

## Abstract

**Background:**

Obesity is now common and this may have altered visual perceptions of what constitutes a ‘normal’ and therefore healthy weight. The present study examined cross-cultural differences in male and female participants’ ability to visually identify the weight status of photographed Caucasian males.

**Methods:**

Five hundred and fifty three male and female young adults from the US (high obesity prevalence), UK and Sweden (lower obesity prevalence) participated in an online study. Participants judged the weight status of a series of photographed healthy weight, overweight and obese (class I) Caucasian males and rated the extent to which they believed each male should consider losing weight.

**Results:**

There was a strong tendency for both male and female participants to underestimate the weight status of the photographed overweight and obese males. Photographed males were frequently perceived as being of healthier weight than they actually were. Some modest cross-cultural differences were also observed; US participants were worse at recognising obesity than UK participants (*p* < 0.05) and were also significantly more likely to believe that the photographed obese males did not need to consider losing weight, in comparison to both the UK and Swedish participants (ps < 0.05). No cross-cultural differences were observed for perceptions or attitudes towards the photographed healthy weight or overweight males.

**Conclusions:**

The weight status of overweight and obese (class I) Caucasian males is underestimated when judged by males and females using visual information alone. This study provides initial evidence of modest cross-cultural differences in attitudes toward, and the ability to recognise, obesity in Caucasian males.

## Background

The prevalence of obesity has grown rapidly in recent times and although obesity is now particularly common in the US [1], forecasts suggest that European countries may develop a similarly high prevalence [2]. This dramatic change to the prevalence of obesity may have also resulted in changes to how heavier body weights are perceived. For example, there is now a very consistent body of literature which indicates that overweight people often underestimate their own weight status, believing that they are not overweight, but instead their weight is ‘about right’ [3, 4]. Moreover this tendency appears to be particularly pronounced amongst males [5, 6]. Studies have also suggested that the degree to which overweight and obese individuals perceive their weight as being ‘normal’ or appropriate is influenced by how common obesity is in their immediate environment [7]. Likewise, recent longitudinal research indicates that although obesity has become more prevalent, the number of overweight and obese individuals correctly identifying their weight status has not increased [8, 9]. These findings suggest that the prevalence of obesity in a region may be an important factor determining whether obesity is recognised and the degree to which individuals feel as though they need to consider addressing their weight. This suggestion has been empirically studied by Wardle and colleagues [10]. In this study, researchers examined self-perceived weight status and weight loss intentions across a number of countries and findings showed that perceived overweight was highest in countries where body weights were generally low (Asian countries), which may indicate that local body weight norms influence perceptions of weight [10].

### Weight misperceptions: self *vs.* others

There is also research which suggests that parents frequently underestimate the weight status of their overweight or obese children [11, 12] and that an increased prevalence of childhood obesity could be partially responsible for this [12]. One interpretation of these findings is that as obesity has become ‘normalised’, overweight and obesity appears less unusual and is therefore harder to visually identify. However, the majority of research to date has focused on either personal weight status misperceptions (lay peoples’ perceptions of their own weight) or parental weight status misperceptions concerning their own children. Because of this, other factors, such as self-serving biases (a bias which enables us to view our own characteristics or those of significant others positively), could in part explain why underestimation occurs. For example, obesity is viewed negatively in the majority of western societies and it is now widely accepted that the overweight and obese are stigmatised [13, 14]. Moreover, some research suggests that this stigmatization may have become worse over time [15]. Because many of us are motivated to want to maintain a positive sense of self [16, 17], it seems reasonable to assume that underestimation of one’s own weight status (or one’s own child’s weight status) could act as a form of self-serving bias. In support of this, individuals with a desire to maintain a slim figure have been shown to be particularly likely to underestimate their weight [18, 19]. Thus, it is difficult to tease apart whether personal weight status misperceptions are caused by visual misperceptions or by self-serving biases. One approach to resolve this issue is to study visual weight status perceptions about *other people,* as individuals should be less motivated to view others in a positive or biased light. Examining visual weight status perceptions of other people allows also for a more direct examination of the ability to visually identify obesity.

### Visual weight status misperceptions

Whether lay people are able to visually recognise overweight and obesity in other people has been examined in some studies [20–23]. In one study it was shown that participants underestimated the weight status of photographed overweight and obese males [22] and other findings suggest that exposure to heavier body weights may be a factor which promotes visual weight status misperceptions [21, 22, 24]. Based on these findings we have theorised that visual perceptions of body weight are malleable and can be shaped by visual input, whereby frequent exposure to obesity may adjust perceptions of what a ‘normal’ weight looks like and this in turn causes visual underestimations of weight status. This interpretation is also in line with the aforementioned epidemiological research which links personal weight status misperceptions to increased obesity prevalence [7–9]. However, there have been relatively few examinations of how common *visual* weight status underestimation is and no studies have examined whether individuals from countries with higher *vs.* low obesity prevalence are more or less likely to underestimate weight status visually.

The aim of the present study was to examine the prevalence of visual weight status misperceptions in female and male adults from three different countries. In the present study we examined male and female participants visual perceptions of male body weight, in order to be able to directly compare our findings to existing studies which have examined visual weight status perceptions [21, 24]. We also reasoned that focusing on male visual weight status perceptions would be valuable, because males have been shown to be far more likely to underestimate their own weight status than females [5, 6]. Thus, visual identification of adiposity in males may be particularly prone to underestimation. In order to examine visual weight status perceptions and attitudes towards males of different body weights, we asked both male and female participants to estimate the weight status of a set of standardised photographs of healthy weight, overweight and obese males. We also asked participants to rate whether they believed each male model needed to consider losing weight, in order to also examine attitudes towards the need for weight loss in males of differing weight statuses. To examine potential cross-cultural differences, we opted to recruit similar cohorts of participants (university students) from the US, UK and Sweden, as the prevalence of male obesity is particularly high in the US; approximately one third [1, 2], in comparison to both the UK and Sweden [25, 26]; approximately one quarter and one fifth respectively. We also recruited both female and male participants, in order to be able to examine potential gender differences in the ability to visually identify adiposity in males.

In line with observations from the personal weight status misperceptions literature, we predicted that participants would tend to underestimate the weight status of overweight and obese males. Moreover, we hypothesised that the country participants were from would impact on weight status misperceptions and attitudes towards males of heavier body weights.

Due to the higher prevalence of obesity in the US, we reasoned that US participants may be more likely to underestimate weight status and also less likely to believe that overweight and obese males needed to consider losing weight, in comparison to both UK and Swedish participants.

## Method

### Participants

In order to obtain similar cohorts of participants from the three different countries we recruited male and female participants using email lists and electronic bulletins from three large universities based in cities in the US (New York), UK (Liverpool) and Sweden (Uppsala) to take part in an online study. The student population in the three universities was predominantly Caucasian English speaking young adults and we did not stratify recruitment. The study was advertised as an online body perception study and there was no compensation offered for participation. The University of Liverpool ethics research committee approved the protocol for the study. Prior to starting the study participants provided informed consent on the study website (tick box). We did not make a formal power calculation, but in order to be comparable with other studies examining visual perceptions of weight status and have a relatively large number of participants from each country, we planned to collect a minimum of 100 participants per country.

### Procedure

Participants first provided demographic information (age, gender ethnicity) and also reported their current weight and height. Participants were then informed that they would be shown 15 photographs of men with varying body mass indices (BMI). They were then shown WHO BMI guidelines for underweight to obese weight statuses (underweight: < 18.5, healthy weight: 18.5-24.9, overweight: 25 – 29.9, obese: 30 kg/m^2^ and above). Photographs were of 18–30 year old Caucasian males standing with their arms at their sides wearing normal fitting short sleeved t-shirts and trousers. We opted to use images of Caucasian males, as that was the ethnicity of the majority of students from the universities we planned to recruit from. For each male two photographs were displayed; one of the male stood front on and one side on, both next to a standardised door frame, in order to provide participants with a point of reference for height and width. There were five healthy weight (BMI M = 21.2, Range 19.4-22.4), five overweight (M = 27.2, Range = 25.7-28.3) and five obese (M = 31.6, Range = 30.5-34.3) models. No information concerning the actual weight status of the participant was presented. None of the models participated in strength building sports or had muscular builds. An earlier pilot study involving male and female UK participants (*n* = 50) rating a wider range of photographed models was conducted in order to select a photograph set of healthy weight, overweight and obese males that were closely matched for attractiveness, height, how muscular they appeared and how tightly fitting their clothes were. For more information about the stimulus set see [20]. Each male was shown on a separate page (in a randomised order) and the central face of each model was obscured with a black box. Underneath each photograph participants were asked to categorise whether they believed the male was ‘underweight’, ‘a healthy weight’, ‘overweight’ or ‘obese’ according to WHO BMI guidelines. Underneath this question participants also rated ‘This person should consider losing weight’ (5 point scale, strongly disagree to strongly agree) for each photograph. On completion participants were thanked for their time and debriefed. The study took approximately 15 min to complete.

### Analysis strategy

#### Perceptions of weight status

In order to examine overall accuracy of perceived weight status, we calculated the number of males (out of 15) that participants correctly categorised (*e.g.* identifying that an obese male was obese). Given that previous research has suggested accuracy may differ dependent on the weight status of the male being categorised [21] we also calculated the number of males (out of 5) correctly categorised for the healthy weight, overweight and obese male photographs individually, before conducting a repeated measures ANOVA with number of males correctly categorised as the dependent variable. In order to test whether country and weight status of photographed males interacted (*e.g.* it might be the case that although participants from the US would be able to identify healthy weight males to a similar degree as UK or Swedish participants, they may be worse at identifying obese males), a follow-up 2×3 ANOVA was used, with country as the between-subjects factor, weight status of photographed males as the within-subjects factor and number of males correctly categorised as the dependent variable.

### Attitudes towards the males’ weight

In order to examine participants’ attitudes towards whether the photographed males needed to consider losing weight, we calculated the mean attitudes score for the five photographs of each weight status category; resulting in separate attitude scores for healthy weight, overweight and obese males. For this analysis we used a 2×3 mixed ANOVA with country as the between-subjects factor, weight status of photographed males as the within-subjects factor and attitudes towards the need for weight loss as the dependent variable.

#### Further investigating effects of country

For weight status perceptions and attitudes, if there was evidence that participant country had any effect in the main ANOVA analyses, we used follow up regression analyses to test whether country differences were still observed when controlling for participant characteristics (*e.g.* age, BMI, gender or ethnicity) which were associated with the dependent variables in question and therefore could be acting as potential confounding variables. We took a conservative approach [27], whereby any demographic variable that was associated with a dependent variable (weight status perceptions or weight loss attitudes) at a *p* ≤ 0.20 was deemed to be a potential confounder and included as a control variable in the regression model. Because our hypotheses concerned differences between US *vs.* UK and Swedish participants, we compared US to UK and Swedish participants in these follow up analyses by creating two dummy coded variables (US *vs.* UK, US *vs.* Swedish) in each regression.

## Results

### Demographics

Of the 553 participants, 182 were from the US, 205 were from the UK and 166 were Swedish. Participants were predominantly Caucasian females (76 % female) aged between 18–30 years old (M = 22.8 years, SD = 5.3) and in the healthy weight range (M BMI = 23.0, SD = 3.7). Although demographics were similar across the three cohorts, there was a significantly higher proportion of males in the Swedish sample than in the US and UK samples (*p* < 0.05) and participants in the UK cohort tended to be slightly younger than the US and Swedish cohorts (*p* < 0.05). See Table 1 for a full breakdown of participant demographics across the three countries.

**Table 1:** US, UK and Swedish participant demographic information

	US (n = 182)	UK (n = 205)	Sweden (n = 166)
Gender (% female)	83.0 % ^a^	78.0 % ^a^	65.1 % ^b^
Age (years; mean and SD)	23.4 (7.5) ^a^	21.7 (3.6) ^b^	23.6 (3.5) ^a^
Self-reported BMI (kg/m^2^; mean and SD)	23.9 (3.9) ^a^ *	22.7 (3.9) ^b^	22.8 (3.1) ^b^
Weight status categories			
Underweight (BMI < 18.5)	2.2 %	9.1 %	6.1 %
Healthy weight (BMI 18.5-24.9)	66.9 %	72.1 %	77.4 %
Overweight (BMI > 24.9 – 29.9)	24.3 %	12.7 %	14.0 %
Obese (Bmi > 29.9)	6.6 %	6.1 %	2.4 %
Ethnicity (% Caucasian)	86.8 %	88.8 %	89.8 %

*A subset of participants entered a particularly large unit for self-reported weights in kilograms (*e.g.* 200 kg). We believe this was due to participants confusing kilograms for pounds. Thus, the BMI data reported in this table is adjusted to exclude these extreme outliers and any participants that did not report full weight or height information

^a,b^ Different characters indicate difference at *p* < 0.05 between countries (as reported in text)

### Perceptions of male weight status across the full sample of participants

We first examined across the full sample whether participants were accurate at categorising the males’ weight status. Out of a total of 15 photographs, the mean number of participants that correctly categorised according to the actual weight status of the photographed male was 5.5 (SD = 2.4). To first examine accuracy of weight status categorisation across the three weight statuses of photographed males (healthy weight, overweight, obese males) for the full sample, a repeated measures ANOVA was used. A main effect was observed (F (1.7, 953.9) = 1070.1, *p* < 0.001, ηp^2^ = 0.67), suggesting that accuracy was dependent on the weight of photographed males being judged. For the 5 healthy weight male photographs M accuracy = 3.5/5 (SD = 1.3), for the 5 overweight male photographs M accuracy = 1.5/5 (SD = 1.3) and for the 5 obese male photographs M accuracy = 0.5/5 (SD = 1.0). All of these scores were significantly different from each other [ts (552) > 19.0, ps < 0.001].

Given that participants tended to be inaccurate, we examined whether inaccuracy was associated with under or overestimation of weight status. For the healthy weight males participants categorised the males as being of a healthy weight the majority of the time (69 % of photographs) and tended to underestimate weight status (30 % of photographs were classed as being underweight) when incorrectly categorising. For the overweight males, participants underestimated weight status (70 % of all photographs) when incorrectly categorising. For the obese males, participants categorised the males as being obese only 10 % of the time, underestimating their weight status as being overweight 67 % of the time and as healthy weight 22 % of the time. See Table 2 for a full breakdown of underestimation by weight status.

**Table 2:** Perceived weight status (in percentages) for the healthy weight, overweight and obese photographed males

Actual weight status	Perceived weight status			
	Underweight	Healthy weight	Overweight	Obese
Healthy weight males	30.1 %	69.3 %	0.6 %	0 %
Overweight males	0.7 %	69.3 %	29.7 %	0.3 %
Obese Males	0.1 %	22.2 %	67.4 %	10.3 %

Values indicate percentage of photographed healthy weight, overweight and obese males (actual weight status) perceived as being underweight, healthy weight, overweight or obese (perceived weight status). 558 participants made 5 perceived weight status observations for each weight status group. Thus, this table represents a total of 8370 observations

### Perceptions of male weight status by country

A 2x3 mixed factor ANOVA was used to compare accuracy across the three weight statuses of photographed males (repeated measures factor: weight status) as a function of country (between subjects factor: country). As in the earlier analysis a main effect of weight status of photographed males was observed [*p* < 0.001]. A main effect of country was observed [F (2, 550) = 0.39, *p* = 0.02, ηp^2^ = 0.01] and the interaction between country and weight status of photographed male was also significant [F(3.5, 952.7) = 2.6, *p* = 0.004, ηp^2^ = 0.01], suggesting that differences in categorisation accuracy across the three weight statues of photographed males was dependent on the country participants were from. We therefore conducted three separate one way ANOVAs with country as a between-subjects factor and number of males accurately categorised for the healthy weight males, overweight males and obese males as the dependent variables. There was no effect of country on the number of healthy weight males participants correctly categorised [F(2, 550) = 1.41, *p* = 0.24, ηp^2^ = 0.005]. However, there was an effect of country for overweight males [F(2, 550) = 3.3, *p* = 0.038, ηp^2^ = 0.01] and for the obese males [F(2, 550) = 6.1, *p* = 0.002, ηp^2^ = 0.02].

As we observed effects of country on the number of overweight and number of obese males accurately categorised, we next planned two forced entry linear regression models (one for overweight male categorization and one for obese male categorization) to test whether any country effects would remain after controlling for participant characteristics. As age (*p* = 0.03) and gender (*p* = 0.02) were associated with the number of obese photographs categorised and BMI (*p* = 0.18) and gender (*p* = 0.08) were associated (at *p* ≤ 0.20) with the number of overweight photos categorised, we included age, gender and BMI in both regression models. Ethnicity was not associated with either the number of overweight males (*p* = 0.31) or obese males (*p* = 0.40) categorised, so was not included in either regression model. We also examined whether ethnicity significantly predicted number of photographs when included as a factor in both regression models and it did not (ps > 0.44).

For the number of overweight males accurately categorised the overall model was significant [F (5, 529) = 2.4, Adjusted R^2^ = 0.017, *p* = 0.02]. Participant gender was a significant predictor (β = 0.09, *p* = 0.049), whereby male participants were more accurate at categorising overweight males than females. However, neither UK (β = 0.07, *p* = 0.18) or Swedish participants (β = 0.08, p = 0.13) differed to the US participants. Participant age and (β = 0.02, p = 0.61) and BMI (β = 0.05, *p* = 0.31) were also non-significant. There was no evidence of significant multi-collinearity (all VIFs < 1.6). For the number of obese males accurately categorised the overall model was significant [F(5, 529) = 4.1, Adjusted R^2^ = 0.03, *p* < 0.001]. Participant gender approached significance (β = 0.08, *p* = 0.06), whereby male participants were more accurate at categorising than females. Participant age was a significant predictor (β = 0.10, *p* = 0.02), whereby older participants tended to be more accurate. Participant BMI was not a significant predictor (β = 0.04, *p* = 0.41). UK participants were more accurate than US participants (β = 0.15, *p* = 0.005), although there was no significant difference between Swedish and US participants (β = −0.04, *p* = 0.48). There was no evidence of significant multi-collinearity (all VIFs < 1.6).

### Attitudes towards the males’ weight across the full sample and by country

**Fig. 1:**
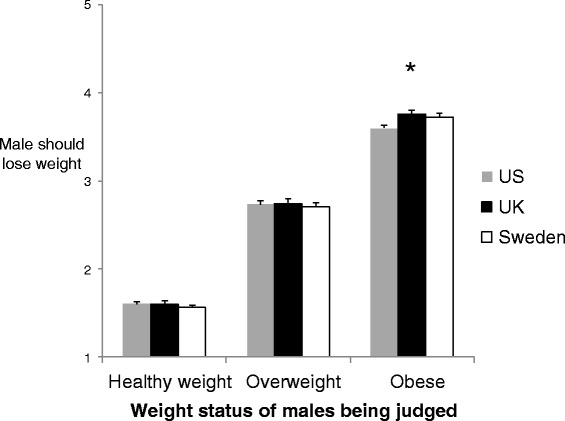
Attitudes towards the need for weight loss of photographed males by country. Higher scores denote greater agreement that male needed to consider losing weight.

A 2×3 mixed factor ANOVA was used to compare attitudes about the need for weight loss across the three weight statuses of photographed males (repeated measures factor: weight status) as a function of country (between subjects factor: country). A main effect of weight status of photographed males was observed [F(1.8, 1008.5) = 3938.5, *p* < 0.001, ηp^2^ = 0.88], whereby males of heavier weight statuses were rated as being in need of weight loss to a greater extent than males of lower weight status. See Fig. 1. No main effect of country was observed [F (2, 550) = 0.43, *p* = 0.65, ηp^2^ = 0.002]. However, there was a small significant interaction between country and weight status of photographed males [F(3.7, 1008.5) = 4.3, p = 0.003, ηp^2^ = 0.02], suggesting that the degree to which participants believed males of different weight statuses needed to lose weight was dependent on country. We therefore conducted three one way ANOVAs with country as a between-subjects factor and attitudes about the need for weight loss in the healthy weight males, overweight males and obese males as the dependent variables. There was no effect of country for the healthy weight [F(2, 550) = 1.3, *p* = 0.26, ηp^2^ = 0.005] or overweight males [F(2, 550) = 0.1, *p* = 0.90, ηp^2^ = 0.001]. However, there was a significant effect of country for the obese males [F(2, 550) = 4.1, p = 0.02, ηp^2^ = 0.02]. As we observed an effect of country on attitudes concerning the need for weight loss in the obese males, we next conducted a forced entry linear regression. Ethnicity was associated with attitudes towards the obese males’ weight (*p* = 0.02) and was therefore including in the model, but age (*p* = 0.88), gender (*p* = 0.37) and BMI (*p* = 0.25) were not, so were not included in the model.

The overall model was significant [F (3, 549) = 4.8, Adjusted R^2^ = 0.02, p = 0.03]. US participants were less likely to believe that the obese males needed to consider losing weight than UK participants (β = 0.14, *p* = 0.006) and less likely than Swedish participants (β = 0.11, *p* = 0.02). Participant ethnicity also had a significant effect (β = 0.10, p = 0.01), whereby Caucasian participants were less likely to believe the obese males needed to consider losing weight. There was no evidence of significant multi-collinearity (all VIFs < 1.4).

## Discussion

The present study examined visual perceptions of male body weight, in female and male participant from the US, UK and Sweden. Across all three countries both male and female participants were poor at visually identifying the weight status of a set of photographed Caucasian males, with participants more often than not believing the photographed overweight and obese males were of a healthier weight status than they actually were. There was also some evidence that UK participants were slightly more accurate at identifying obesity than US participants, although there were no cross-cultural differences when perceiving the weight status of healthy weight or overweight males. A similar pattern of results was observed when examining participant attitudes towards the need for weight loss for the overweight and obese males. Regardless of country, participants tended not to believe that the overweight and obese males needed to consider losing weight. However, US participants were slightly less likely to believe that the obese males needed to consider losing weight, in comparison to both the UK and Swedish participants.

This is the first study we are aware of to specifically examine visual perceptions of male Caucasian weight status in countries with differing obesity prevalence. The findings of this study are similar to those which have tested whether visual recognition of weight status is accurate in person. For example, a number of studies have tended to show that when judging the weight of other people, medical professionals’ accuracy is poor and they often underestimate weight status [28–30]. Our results are also in line with recent suggestions that weight status is visually underestimated [20–21], participants tended to visually perceive the overweight and obese males as being of healthier weight than they actually were. We also observed some modest cross-cultural differences. Participants from a country in which obesity is particularly prevalent (US), were more likely to visually underestimate the weight status of obese males and more likely to believe that the obese males did not need to consider losing weight. Although these cross-cultural differences were relatively small in size, they appear to support recent experimental evidence indicating that exposure to heavier body weights may result in visual weight status perceptions and increased acceptance of heavier body weights [22, 24]. We suggest the observed cross-country differences in the present study may be a consequence of the higher obesity prevalence in the US, compared to the UK and Sweden. However, the country effects observed were statistically small in the present study and our proposed interpretation is speculative, so further work designed to replicate and examine the specific mechanisms causing cross-cultural differences is now needed. It may also be the case that larger cross-cultural differences would have been observed if we had sampled some countries with very low male obesity prevalence (*e.g.* < 10 %).

### Underestimating weight status

The present study focused on visual weight status perceptions of males. Epidemiological work has indicated that overweight and obese males are significantly more likely to underestimate their own weight status than healthy weight males [5, 6]. The results of the present study concerning visual perceptions of weight status effectively mirror this finding; underestimation of weight status of the photographed overweight and obese males occurred more frequently than underestimation of the healthy weight males. These findings indicate that both male *personal* weight status perceptions and *visual* weight status perceptions of male body weight are prone to underestimation. Regardless of participant country, gender, ethnicity or age, on the whole participants tended to be quite accurate when estimating the weight status of healthy weight males, but as weight status increased so did underestimation, such that obese males were only accurately categorised approximately 10 % of the time. These findings are in line with a recent study examining female participants’ perceptions of female body weight [23], which showed that African American Women were poor at identifying overweight female body figures, suggesting that visual perceptions of weight status differ to medical definitions of weight status.

### Implications of visually underestimating weight status?

An interesting question is whether there are implications of visually underestimating weight status? One possibility is that a tendency to visually underestimate weight may increase the likelihood that an individual misperceives their own weight or the weight of those around them (*e.g.* a spouse or child). Thus, underestimation could result in individuals being less likely to believe their weight needs modifying [31], which in turn could make them less likely to attempt weight loss or encourage healthier weight related behaviours in others [32]. However, it may also be the case that ignorance is bliss [33]. For example, because obesity is now stigmatised, underestimating your own weight status could in theory be protective to psychological well-being [33] and reduce unhealthy weight control behaviours [34] amongst individuals who are overweight. Further work examining whether underestimation of weight status is associated with personal weight gain or a reduced likelihood of motivating others who are overweight or obese to consider addressing their weight, would now be informative.

### Visual perceptions of female weight status

A significant limitation of the present research is that examination of visual weight status perceptions of females was outside of its scope, so we do not know whether similar findings would be observed when judging female weight status. We reason that it is possible a different pattern of results could be observed when judging female body weight, so future work examining this would now be informative. For example, female body weight is often judged more critically than male weight [13], so it might be the case that underestimation of weight status would be less pronounced, or even in some cases female weight status may be visually overestimated.

### Strengths & limitations

In the present study we used a standardised photograph set of males of different weight statuses. Although males were shown both front and side on, it may be the case that weight status perceptions would have been generally more accurate if the males were shown in 3D or judged in person. Yet, this limitation seems less likely to explain the differences in underestimation across weight statuses and the observed cross-country effects. The photographed obese males in the present study were of class-one obesity. Obese males of heavier body weights would presumably be easier to visually identify as obese. A strength of the present work was the use of standardised images of real males of different body weights, as other research has often relied on silhouette drawings or computer simulated avatars (*e.g.* see [35]), which can be criticised for not accurately capturing obesity in humans. A limitation was that we mainly sampled female Caucasians. Because of this we cannot generalise our findings to individuals of different ethnicities, as there is some research suggesting that there are ethnic differences in body size attitudes (*e.g.* [35]). Another issue with our sample is that they were mainly female and likely to be of relatively high socio-economic status (SES), compared to the general population. We found no evidence of gender effects on visual identification of male overweight, but SES may have relevance to the cross-cultural differences we observed. As there has been some research indicating that SES is associated with differences in ideal body size [36], it would have been valuable to have measured the SES of participants in the present study. Although we recruited cohorts of young adults from similar SES backgrounds (and identical in terms of level of education), by directly measuring SES this would have allowed us to directly test whether the cross-cultural differences we observed could have been in part attributed to SES. For example, individuals of low SES may be more likely to encounter obesity on a day to day basis [37], which in turn could increase the likelihood that they would visually underestimate weight status.

### Public health application

The present findings are in line with suggestions that as a society, we may have a poor understanding of what (male) overweight body weights look like [21]. One implication of this observation is that it may be necessary to design interventions which educate members of the general public and correct perceptions of what body sizes normally constitute a ‘healthy’ weight. However, such public health approaches will need to be sensitive to the stigma attached to being overweight.

## Conclusions

The weight status of overweight and obese (class I) Caucasian males is underestimated when judged using visual information alone. This study provides initial evidence of modest cross-cultural differences in attitudes toward, and the ability to recognise, obesity in Caucasian males.
